# Enhancement of P2X3 Receptor-Mediated Currents by Lysophosphatidic Acid in Rat Primary Sensory Neurons

**DOI:** 10.3389/fphar.2022.928647

**Published:** 2022-06-20

**Authors:** Wen-Long Qiao, Qing Li, Jia-Wei Hao, Shuang Wei, Xue-Mei Li, Ting-Ting Liu, Chun-Yu Qiu, Wang-Ping Hu

**Affiliations:** ^1^ School of Basic Medical Sciences, Xianning Medical College, Hubei University of Science and Technology, Xianning, China; ^2^ School of Pharmacy, Xianning Medical College, Hubei University of Science and Technology, Xianning, China; ^3^ Hubei College of Chinese Medicine, Jingzhou, China

**Keywords:** lysophosphatidic acid, P2X3 receptor, current, dorsal root ganglion neuron, nociceptive behavior

## Abstract

Lysophosphatidic acid (LPA), a lipid metabolite, plays a role in both neuropathic and inflammatory pain through LPA_1_ receptors. P2X3 receptor has also been shown to participate in these pathological processes. However, it is still unclear whether there is a link between LPA signaling and P2X3 receptors in pain. Herein, we show that a functional interaction between them in rat dorsal root ganglia (DRG) neurons. Pretreatment of LPA concentration-dependently enhanced α,β-methylene-ATP (α,β-meATP)-induced inward currents mediated by P2X3 receptors. LPA significantly increased the maximal current response of α,β-meATP, showing an upward shift of the concentration-response curve for α,β-meATP. The LPA enhancement was independent on the clamping-voltage. Enhancement of P2X3 receptor-mediated currents by LPA was prevented by the LPA_1_ receptor antagonist Ki16198, but not by the LPA_2_ receptor antagonist H2L5185303. The LPA-induced potentiation was also attenuated by intracellular dialysis of either G-protein inhibitor or protein kinase C (PKC) inhibitor, but not by Rho inhibitor. Moreover, LPA significantly changed the membrane potential depolarization and action potential burst induced by α,β-meATP in DRG neurons. Finally, LPA exacerbated α,β-meATP- induced nociceptive behaviors in rats. These results suggested that LPA potentiated the functional activity of P2X3 receptors in rat primary sensory neurons through activation of the LPA_1_ receptor and its downstream PKC rather than Rho signaling pathway, indicating a novel peripheral mechanism underlying the sensitization of pain.

## Introduction

Lysophosphatidic acid (LPA), a lipid metabolite, is further released under various pathological states such as tissue injury and inflammation (Eichholtz et al., 1993). LPA participates in a variety of cellular processes, such as cell growth, proliferation, migration, morphogenesis, differentiation and survival (Yung et al., 2014). LPA exerts its biological effects by activating six specific G protein-coupled receptors (GPCRs), named LPA_1-6_ receptors, and downstream multiple signaling pathways (Anliker and Chun, 2004; Yung et al., 2014). LPA also plays an important role in both neuropathic and inflammatory pain through LPA_1_ receptors (Inoue et al., 2004; Ueda, 2008; Velasco et al., 2017; Srikanth et al., 2018). The LPA_1_ receptor antagonist Ki-16425 has shown efficacy in relief of neuropathic pain (Ma et al., 2009). Neuropathic pain is attenuated in LPA_1_ receptor-deficient mice, indicating involvement of LPA signaling through LPA_1_ receptors (Inoue et al., 2004). Among all LPA receptors, LPA_1_ subtype is mainly expressed in DRG neurons (Inoue et al., 2004). LPA_1_ receptor is coupled to four distinct G_α_ proteins and triggers multiple intracellular signaling pathways, which may be involved in the modulation of the expression and function of some pain-related ion channels by LPA (Anliker and Chun, 2004; Yung et al., 2014; Hernandez-Araiza et al., 2018; Juarez-Contreras et al., 2018). For example, LPA_1_ receptors activate the Rho signaling by coupling to G_12/13_ proteins, resulting in an increase in the expression of Ca_v_3.2 and Ca_v_α2δ1 channels (Inoue et al., 2004; Juarez-Contreras et al., 2018). Furthermore, LPA_1_ receptor is coupled to G_q/11_, up-regulating Nav1.8 expression and potentiating Nav1.8 currents in DRG neurons (Seung Lee et al., 2005; Pan et al., 2016). Electrophysiological experiments have shown LPA_1_ receptor activation and intracellular PKC signaling are crucial for transient receptor potential V1 (TRPV1) sensitization by LPA (Pan et al., 2010). Therefore, targeting these ion channels is the key for LPA to participate in pain.

P2X3 receptor is also an important pain-related ion channel, which is expressed in primary sensory neurons, including the ends of nociceptive fibers. P2X3 receptor has been shown to participate in multiple pain processes, including neuropathic and inflammatory pain (Burnstock, 2016). For example, Antagonists and antisense oligonucleotide of P2X3 receptors are effective in pain relief (Honore et al., 2002; Jarvis et al., 2002; McGaraughty et al., 2003). Administration of ATP or formalin causes attenuated spontaneous pain behaviors in mice lacking P2X3 receptor (Cockayne et al., 2000; Souslova et al., 2000). Increased ATP currents and aggravated pain evoked by P2X3 receptor activation are observed under nerve injury and inflammation conditions (Hamilton et al., 2001; McGaraughty et al., 2003; Xiang et al., 2008). In rats with bone cancer, the expression of LPA_1_ receptors is increased in DRG neurons (Wu et al., 2016). Furthermore, activation of LPA_1_ receptors by LPA up-regulates the expression of P2X3 receptors *via* a Rho-ROCK pathway in bone cancer pain (Wu et al., 2016). In addition to the expression of P2X3 receptors, it was still unclear whether LPA/LPA_1_ receptor signaling enhances the functional activity of P2X3 receptors. The present study shows that activation of LPA_1_ receptors by LPA potentiated the electrophysiological activity mediated by P2X3 receptors in rat DRG neurons *via* an intracellular PKC rather than Rho signaling pathway, including potentiation of P2X3 receptor-mediated ATP currents and membrane excitability. LPA also exacerbated nociceptive behaviors mediated by P2X3 receptors in rats through peripheral LPA_1_ receptors.

## Materials and Methods

### Preparation of DRG Neurons

All experimental protocols were approved by the Animal Research Ethics Committee of Hubei University of Science and Technology (2016-03-005). Sprague-Dawley rats (5–6 weeks old) were sacrificed after anesthesia. The DRGs of lumbar segments 4–6 were taken out and cut into pieces. The chopped ganglia were transferred to a tube containing Dulbecco’s modified Eagle’s medium (DMEM) and incubated with shaking for 25–30 min at 35°C. The incubation solution contained trypsin (0.5 mg/ml), collagenase (1.0 mg/ml) and IV DNase (0.1 mg/ml). Soybean trypsin inhibitor (1.25 mg/ml) was added to stop trypsin digestion. These cells were cultured in DMEM containing 10% fetal bovine serum and 100 ng/ml of nerve growth factor for 12–24 h at 37°C.

### Electrophysiological Recordings

As described previously (Hao et al., 2022), whole-cell patch-clamp recordings were carried out using A MultiClamp-700B amplifier and Digidata-1550B A/D converter (Axon Instruments, CA, United States). Before electrophysiological recordings, the prepared DRG cells were maintained in 35 mm dish filled with normal external solution for at least 60 min. The external solution contained the following (in mM): 150 NaCl, 2.5 CaCl_2_, 5 KCl, 2 MgCl_2_, 10 HEPES, and 10 D-glucose. The pH was adjusted to 7.4 with NaOH, and the osmolarity was adjusted to 330 mOsm/L with sucrose. The recording micrepipettes were pulled using a Sutter P-97 puller (Sutter Instruments, CA, United States), whose resistance was 3–6 MΩ. The micropipette solution contained the following (in mM): 140 KCl, 2 MgCl_2_, 10 HEPES, 11 EGTA, 4 ATP, and 0.3Na_2_GTP. The pH and osmolarity were adjusted to 7.2 with KOH and 310 mOsm/L with sucrose, respectively. After gigaseal formation the pipette capacitance mediated current transients were compensated, and then the membrane beneath the pipette was ruptured by suction to form whole cell recording configuration. Then whole cell capacitance compensation was done, after which series resistance (Rs) was compensated by 70–80%. Rs was re-checked at the end of the recordings, and the data with Rs variation exceeding 20% were discarded. The recorded currents were low-pass filtered at 2 kHz, and sampled at 10 kHz. Only small- and medium-sized nociceptive DRG cells (15–40 μm in diameter) were used for the electrophysiological recordings. The membrane potential of the recorded cells was clamped at -60 mV unless otherwise stated. The liquid junction potential was calculated at 4 mV, which, being a DC voltage small enough compared with the driving force for ATP currents, was omitted. Current-clamp recordings were carried out in only DRG cells whose resting membrane potentials were less than -50 mV.

### Drug Application

All drugs were purchased from Sigma-Aldrich (St. Louis, MO, United States) and freshly prepared to working concentration in normal external solution. Each drug was stored in different reservoirs and subjected to gravity. The distance between the recorded cell and the drug exit was approximately 30 μm. The internal solution containing antagonist or blocker were delivered intracellularly through the recording micropipettes to block G-proteins and intracellular signaling as described previously (Qiu et al., 2012). To ensure that these drugs were fully infused into the tested cell, current recordings were carried out at least 30 min after cell membrane rupture.

### Nociceptive Behavior Induced by α,β-meATP in Rats

Rats were habituated for 30 min in a Plexiglas chamber before the nociceptive behavioral test. The rats in six different groups received 50 μl intraplantar injections of vehicle, different doses (0.2, 2 and 20 ng) of LPA, 60 ng Ki16198 + 20 ng LPA, or 60 ng H2L5185303 + 20 ng LPA, respectively. After 10 min, α,β-methylene-ATP (α,β-meATP, 50 μg in 50 μl) was injected into the ipsilateral hindpaws and tested the nociceptive behaviors by other experimenters. The assessor of the behavioral measures was blinded to the prior treatment conditions. In the other group, rats received 50 μl intraplantar injections of 20 ng LPA alone. The nociceptive behaviors (that is, number of flinches) were monitored within 10 min after α,β-meATP injection. Meanwhile, mechanical allodynia was measured by paw withdrawal threshold (PWT). PWT of the ipsilateral hind plantar using a series of von Frey filaments (Stoelting, Wood Dale, IL) at 0.5, 2.5,5, and 24 h after α,β-meATP injection. Rats received intrathecal injection of drugs by lumber puncture. Briefly, rats were anaesthetized with isofluorane in a transparent plastic box and then placed on a roller so that the L4-6 vertebrae were curved. A lumbar puncture needle was introduced into the intrathecal space. The needle had been introduced intrathecally when a short flicking of the tail was observed. Then drugs were slowly injected into the intrathecal space. The needle was immediately pulled out after the injection.

### Data Analysis

Student’s t-test and analysis of variance (ANOVA) followed by Bonferroni’s *post hoc* test were used to analyze the experimental data, which were expressed as mean ± S.E.M. The concentration–response data were analyzed using the non-linear curve-fitting program ALLFIT.

## Results

### LPA Concentration-dependently Enhances ATP Currents in Rat DRG Neurons

**FIGURE 1:**
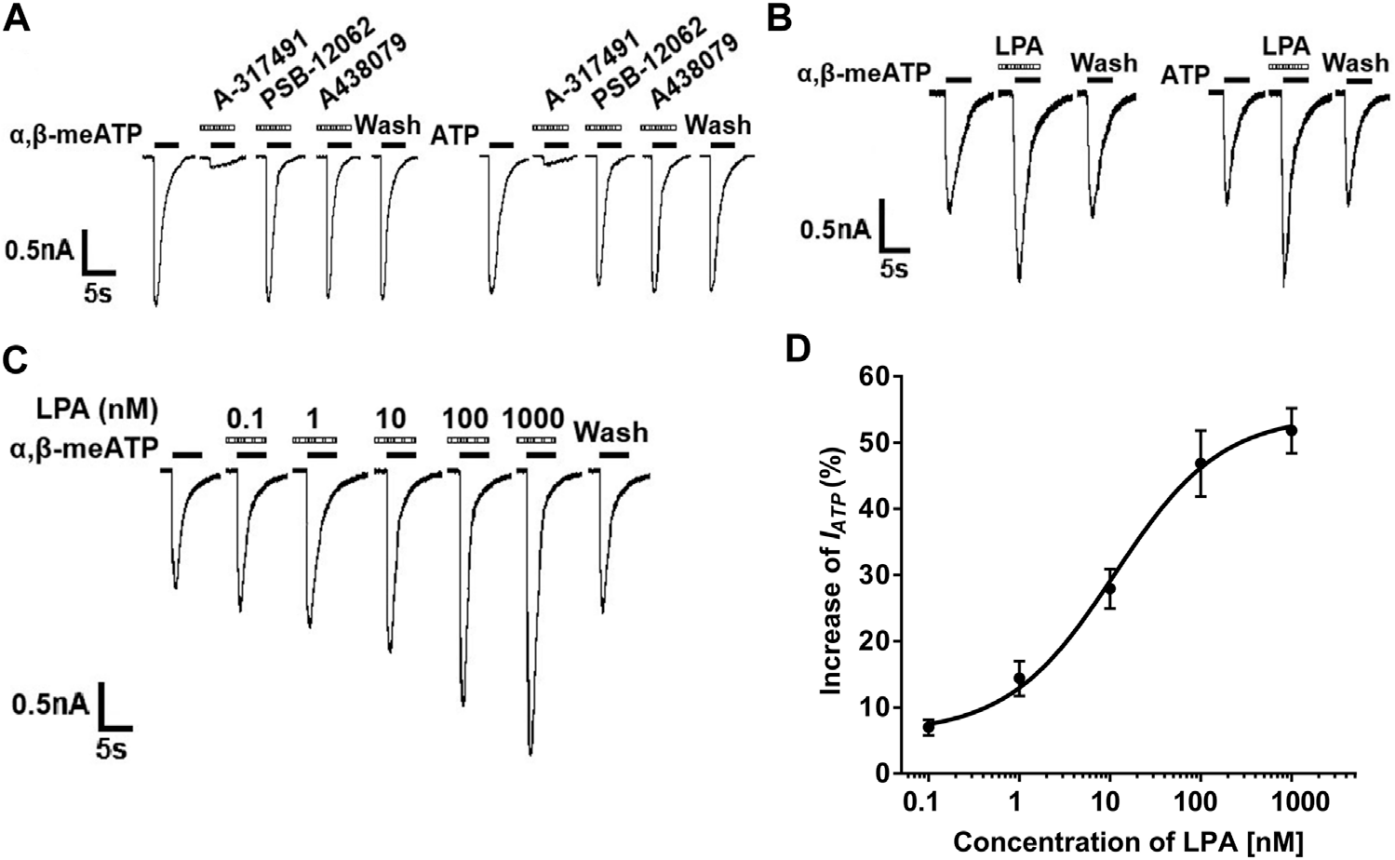
LPA-induced potentiation of P2X3 receptor-mediated ATP currents in DRG neurons. **(A)** Both α,β-meATP (30 μM) and ATP (30 μM) can produce inward currents in the same DRG cell, which were blocked by the P2X3 and P2X2/3 receptor antagonist A-317491 (100 μM), but not by the P2X4 receptor antagonist PSB-12062 (10 μM) and the P2X7 receptor antagonist A438079 (1 μM). Membrane potentials were clamped at −60 mV. **(B)** The 30 μM α,β-meATP- and ATP- induced currents were similarly enhanced by pre-application of LPA (100 nM for 5 min) to a DRG cell. **(C)** The sequential current traces illustrate that a gradual increase in the I_ATP_ amplitude with an increase in LPA concentration from 0.1 to 1,000 nM in a recorded DRG neuron. **(D)** The graph shows the concentration-effect curve of LPA on I_ATP_ with an EC_50_ value of 11.18 ± 0.58 nM. I_ATP_ was evoked by 30 μM α,β-meATP. Each point represents the mean ± S.E.M. of 6–8 cells.

In most DRG cells (70.0%, 7/10), α,β-methylene-ATP (α,β-meATP, 30 μM) or ATP (30 μM) can evoke a rapid inward currents (I_ATP_) at holding potentials of −60 mV (Figure 1A). These ATP currents were completely blocked by 100 μM A-317491, a specifical P2X3 and P2X2/3 receptor antagonist. In contrast, PSB-12062 (a P2X4 receptor antagonist, 10 μM) and A438079 (a P2X7 receptor antagonist, 1 μM) had no effect on I_ATP_. these results indicated that these ATP currents were mediated by P2X3 or P2X2/3 receptors. In all ATP currents recorded, we observed that three type ATP currents: a fast transient current, a slow transient inward current, and an intermediate type current. LPA similarly enhanced the three type ATP currents. LPA similarly enhanced the three ATP currents. In order to purify the type of ATP current, we mainly observed the effect of LPA on this fast transient ATP currents in the following experiments.

In most DRG neurons tested (72.7%, 8/11), we observed that the α,β-meATP- and ATP- evoked currents were increased when 100 nM LPA was pre-treated to DRG cells for 5min prior to the next recording (Figure 1B). The increase of I_ATP_ depended on the concentration of LPA. The sequential current traces in Figure 1C illustrated that a gradual increase in the I_ATP_ amplitude with an increase in LPA concentration from 0.1 to 1,000 nM in a recorded DRG neuron. The EC_50_ (half-maximal effective concentration) value was 11.18 ± 0.58 nM according to concentration-effect curve of LPA on 30 μM α,β-meATP evoked I_ATP_ in Figure 1D. These results suggest that LPA concentration-dependently enhanced P2X3 receptor-mediated ATP currents.

### Concentration–Response and Current–Voltage Relationships for α,β-meATP With and Without LPA Pretreatment

**FIGURE 2:**
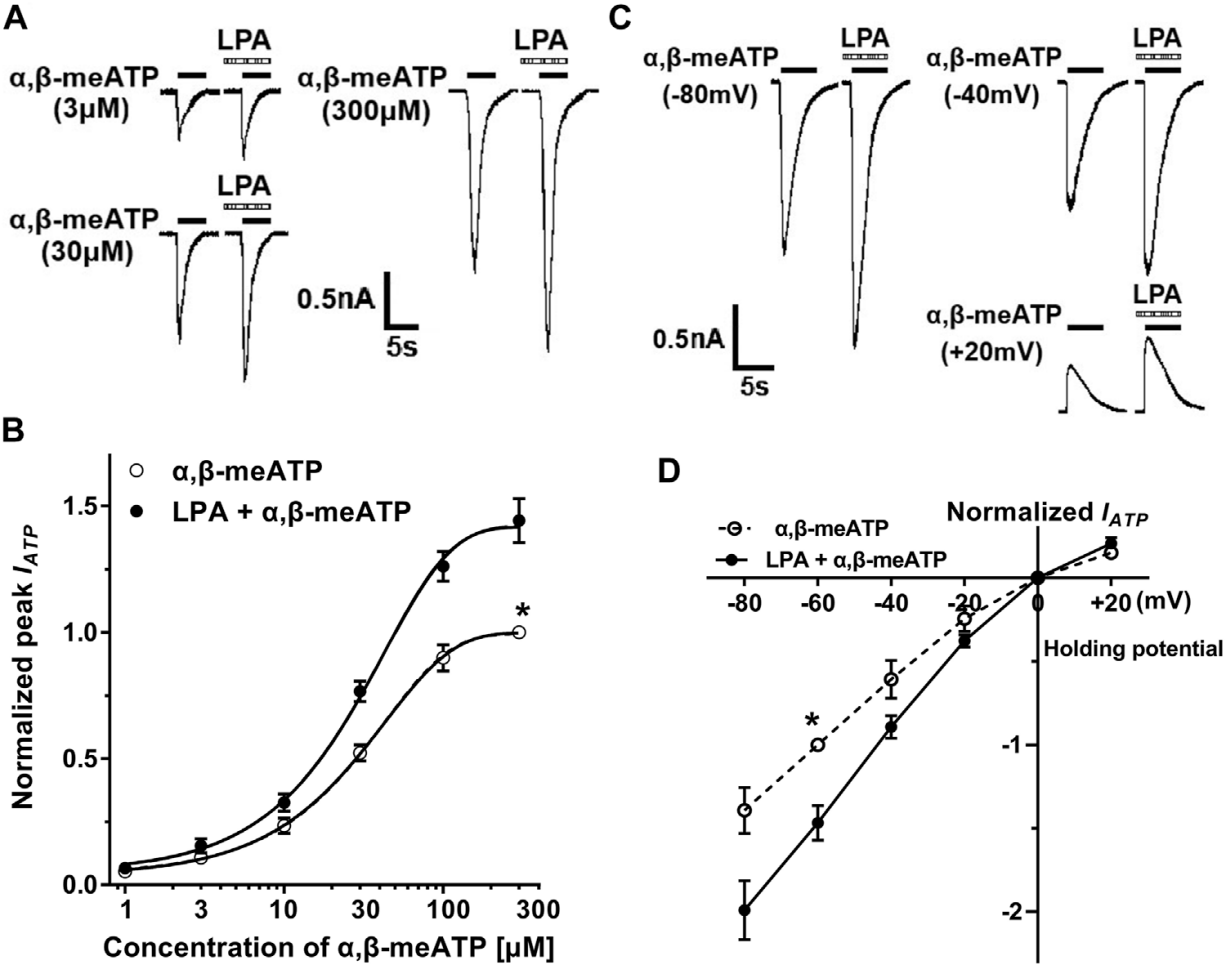
Concentration–response and current–voltage (I–V) relationships for α,β-meATP with and without LPA pretreatment. **(A)** The sequential current traces illustrate the effects of pre-application of LPA (100 nM for 5 min) on three representative ATP currents evoked by 3, 30 and 300 μM α,β-meATP, respectively. **(B)** LPA (100 nM) pretreatment shifted upwards the concentration-response curve for α,β-meATP. Each point represents the mean ± S.E.M. of 6–8 cells. Each current value from the same neuron was normalized to the current response evoked by α,β-meATP (300 μM) without LPA pretreatment. **(C)** The sequential current traces illustrate that pre-application of LPA (100 nM for 5 min) increased currents evoked by 30 μM α,β-meATP at holding potentials of -80, -40, and +20 mV, respectively. **(D)** The I–V curves for 30 μM α,β-meATP-induced currents (I_ATP_) in the absence and presence of LPA (100 nM). Each current value from the same neuron was normalized to the current response evoked by α,β-meATP (300 μM) without LPA pretreatment at holding potential of −60 mV. Each point represents the mean ± S.E.M. of 6–8 cells. The recording micropipettes were filled with an internal solution containing CsCl.

We observed whether the effect of LPA on ATP currents depended on concentration of α,β-meATP. Figure 2A shows that pre-application of LPA (100 nM for 5 min) increased the amplitudes of I_ATP_ evoked by 3, 30 and 300 μM α,β-meATP, respectively. The concentration-response curves in Figure 2B were drawn using a series of α,β-meATP concentration in the absence and presence of LPA (100 nM), which were fit with the Hill equation. LPA pretreatment shifted upwards the concentration-response curve for α,β-meATP with an increase of 44.18 ± 8.74% in α,β-meATP (300 μM) -induced maximal current response (*p* < 0.01, Bonferroni’s *post hoc* test). However, the Hill coefficients or the slopes were not significantly different between the two curves, which were 1.48 ± 0.25 and 1.56 ± 0.28, respectively, in the absence and presence of LPA (*p* > 0.1, Bonferroni’s *post hoc* test). In addition, LPA had no effect on the EC_50_ of α,β-meATP, which were 28.26 ± 1.68 μM without LPA pretreatment and 27.88 ± 1.74 μM with LPA pretreatment, respectively (*p* > 0.1, Bonferroni’s *post hoc* test). These results suggest that the maximum response to α,β-meATP, but not affinity, was modulated by LPA.

We then investigated the effect of LPA on ATP currents recorded at different clamping potentials. Figure 2C shows that LPA pretreatment (100 nM for 5 min) increased I_ATP_ amplitude evoked by 30 μM α,β-meATP in a DRG cell with clamping potentials at −80, −40 and +20 mV, respectively. Current–voltage (I–V) curves for α,β-meATP in Figure 2D were drawn using a series of clamping potentials in the absence and presence of LPA (100 nM). The enhancement of ATP currents by LPA was not significantly different at clamping potentials from −80 to 20 mV (*p* > 0.1, Bonferroni’s *post hoc* test), showing an increased slope of I–V curve with LPA pretreatment. LPA had no effect on the reversal potential (close to 0 mV). These results indicate that enhancement of ATP currents by LPA was voltage independent.

### LPA Enhances ATP Currents *via* a LPA_1_ Receptors, G_q/11_-Proteins and PKC Signaling Pathway

**FIGURE 3:**
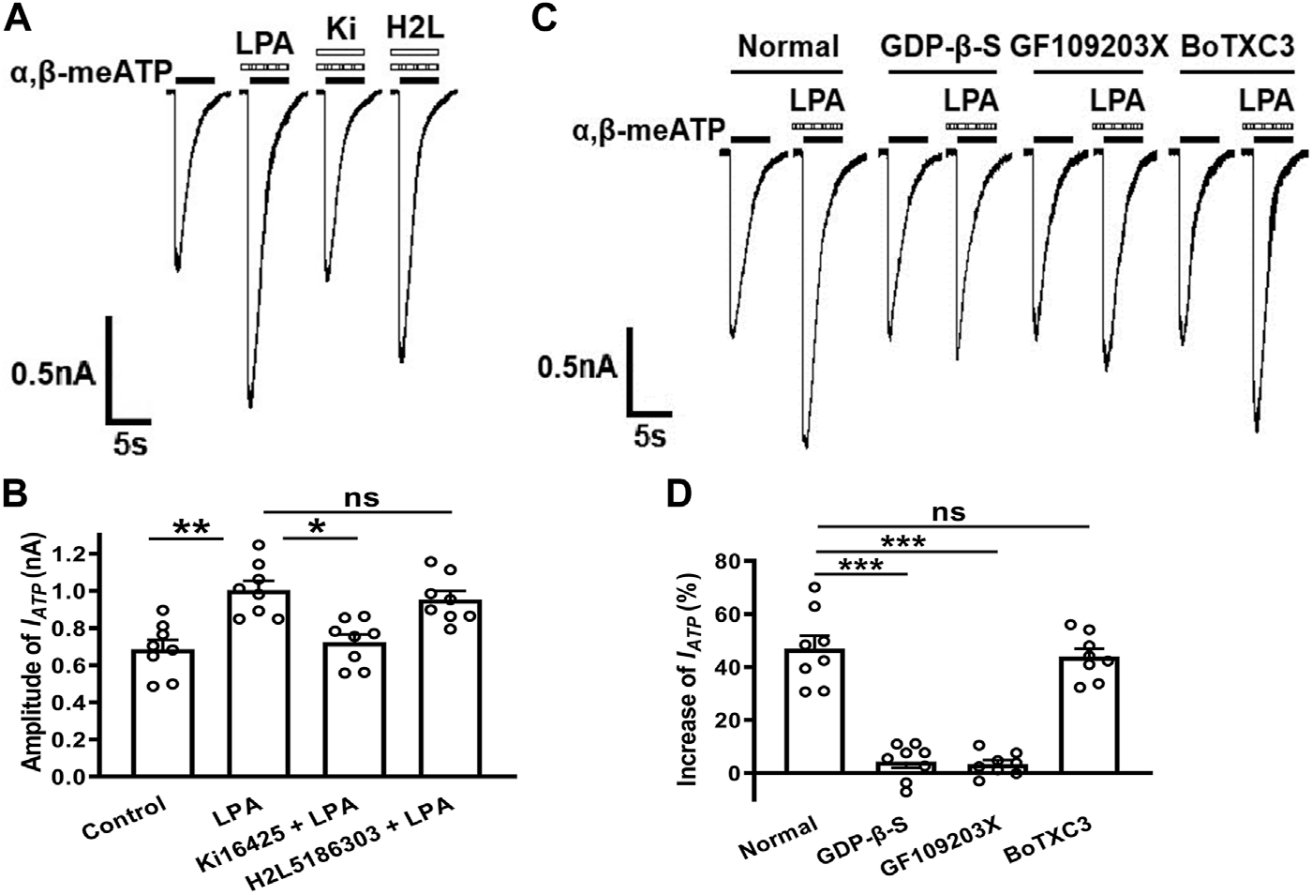
Involvement of LPA_1_ receptors, G_q/11_-proteins and PKC signaling in the LPA-evoked potentiation of ATP currents. Representative current traces in **(A)** and the bar graph in **(B)** show that I_ATP_ was enhanced by pre-application of 100 nM LPA alone for 5 min in DRG cells. The LPA-induced enhancement was prevented by co-treatment of the LPA_1_ receptor antagonist Ki16198 (300 nM), but not by the LPA_2_ receptor antagonist H2L5185303 (300 nM). *p < 0.05, **p < 0.01; ns, No significant; Bonferroni’s *post hoc* test, *n* = 8 in each column. The current traces in **(C)** and the bar graph in **(D)** show that LPA (100 nM) had little effect on I_ATP_ in the recording micropipettes filled with an internal solution containing the non-hydrolyzable GDP analog GDP-β-S (500 μM), or the PKC inhibitor GF109203X (2 μM), which was different from the enhancing effect under normal internal solution conditions. ***p < 0.001, Bonferroni’s *post hoc* test, compared with normal column. *n* = 8 in each column. However, LPA (100 nM) had a similar enhancing effect on I_ATP_ under internal solution containing the Rho inhibitor BoTXC3 (5 pg/μl) conditions. ns, No significant.

To identify whether the LPA receptors mediate LPA enhancement of ATP currents, LPA_1_ and LPA_2_ receptor antagonists were co-treated with LPA to recorded cells. As shown in Figures 3A,B, LPA (100 nM) pre-treatment increased I_ATP_ amplitude from 0.69 ± 0.05 nA to 1.01 ± 0.07 nA in 8 tested cells (*p* < 0.01, one-way ANOVA followed by *post hoc* Bonferroni’s test). However, the I_ATP_ amplitude was 0.72 ± 0.04 nA when 100 nM LPA was co-treated with 300 nM Ki16198, which was significantly different from the I_ATP_ amplitude of 1.01 ± 0.07 nA after LPA pretreatment alone (*p* < 0.05, one-way ANOVA followed by *post hoc* Bonferroni’s test, *n* = 8), suggesting LPA-induced potentiation of I_ATP_ was blocked by the LPA_1_ receptor antagonist Ki16198. When 100 nM LPA was co-treated with 300 nM H2L5185303, a LPA_2_ receptor antagonist, the I_ATP_ amplitude was 0.96 ± 0.04 nA, which was not significantly different from the I_ATP_ amplitude after LPA pretreatment alone. These results indicate that LPA enhanced ATP currents through LPA_1_ receptors, but not LPA_2_ receptors.

LPA_1_ receptor, a member of GPCR family, can initiate intracellular events once activated (Anliker and Chun, 2004; Yung et al., 2014). We then identified which intracellular signaling of LPA_1_ receptor activation contributed to the potentiation of ATP current by LPA. First, GDP-β-S (a non-hydrolyzable GDP analog, 500 μM) was dialyzed intracellularly into DRG neurons through the recording micropipettes before LPA pretreatment. Unlike an increase of 46.37 ± 4.97% induced by 100 nM LPA in I_ATP_ amplitude under the normal internal solution conditions, LPA (100 nM) pretreatment failed to increase I_ATP_ in cells treated with GDP-β-S (Figures 3C,D). Second, we further explored the intracellular signaling involved in LPA-induced enhancement. GF109203X (a selective PKC inhibitor, 2 μM) was contained in the intracellular solution. LPA (100 nM) pretreatment also failed to increase I_ATP_ in GF109203X-treated cells. Last, BoTXC3 (a Rho inhibitor by ADP-riboslation, 5 pg/μL) was delivered intracellularly, LPA (100 nM) pretreatment still produced an increase of 43.94 ± 3.05% in I_ATP_ amplitude, suggesting that Rho signaling may not be important for LPA to regulate P2X3 receptors (Figures 3C,D). These data collectively indicate that potentiation of ATP currents by LPA depended on a GPCR and downstream PKC rather than Rho signaling pathway.

### LPA Increases α,β-meATP -Induced Membrane Excitability of Rat DRG Neurons

**FIGURE 4:**
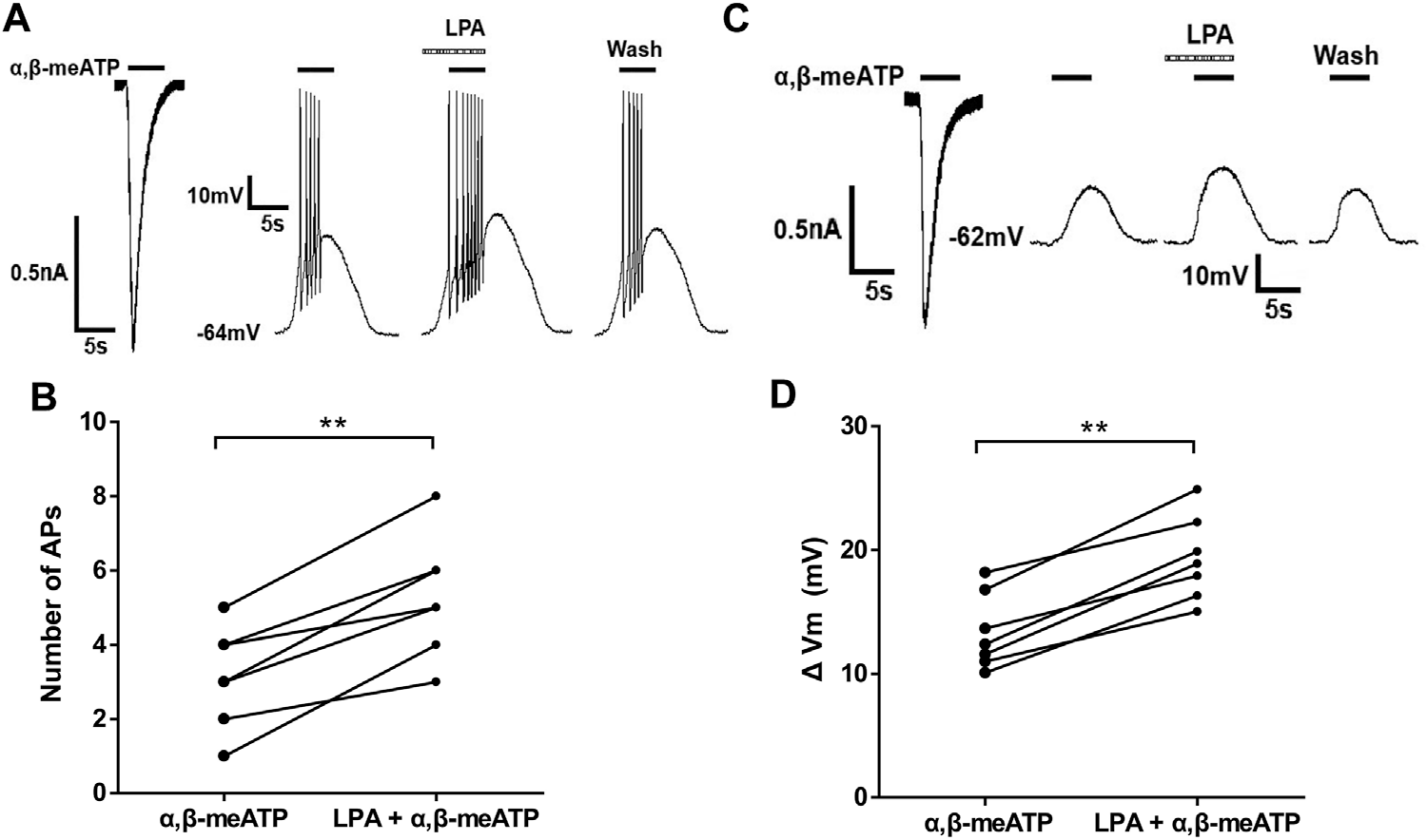
LPA-induced enhancement of membrane excitability induced by α,β-meATP in DRG cells. Original traces in **(A)** show that α,β-meATP (30 μM) produced a current and action potentials (APs) under voltage- and current-clamp conditions, respectively, Original traces in **(B)** show that α,β-meATP (30 μM) produced a current and membrane depolarization in the presence of TTX (1 μM) to block Na^+^ channel-mediated APs under voltage- and current-clamp conditions, respectively, in the same DRG neuron. The number of α,β-meATP-induced APs in **(C)** and membrane depolarization (ΔVm) in **(D)** were quantified before and after LPA treatment (100 nM for 5 min) in seven DRG neurons. **p < 0.01, paired *t*-test, *n* = 7 cells.

We then observed whether LPA had an effect on α,β-meATP-evoked membrane excitability. Figures 4A,B show that α,β-meATP (30 μM) induced an inward current and also produced action potentials (APs) in the same DRG neurons under voltage-clamp and current-clamp conditions, respectively. Pre-application of LPA (100 nM for 5 min) increased the number of α,β-meATP-evoked APs, consistent with effect of LPA in voltage-clamp recordings. The combined data in Figure 4B show that the number of APs increased from 3.14 ± 0.51 of control condition to 5.29 ± 0.61 of LPA pre-treatment in the seven neurons (*p* < 0.01, paired t-test, *n* = 7).

To investigate the effect of LPA on α,β-meATP-evoked membrane depolarization, TTX (1 μM) was used to block Na^+^ channel-mediated APs. As shown in Figure 4C, pre-application of LPA (100 nM for 5 min) enlarged the depolarization evoked by 30 μM α,β-meATP. In seven neurons, the magnitude of depolarization (ΔVm) increased from 13.38 ± 1.15 mV to 19.31 ± 1.29 mV after 100 nM LPA pretreatment for 5 min (paired t-test, *p* < 0.01, *n* = 7; Figure 4D). These results suggest that LPA increased α,β-meATP-induced membrane excitability of rat DRG cells.

### PLA Exacerbates α,β-meATP-Evoked Nociceptive Behaviors in Rats

**FIGURE 5:**
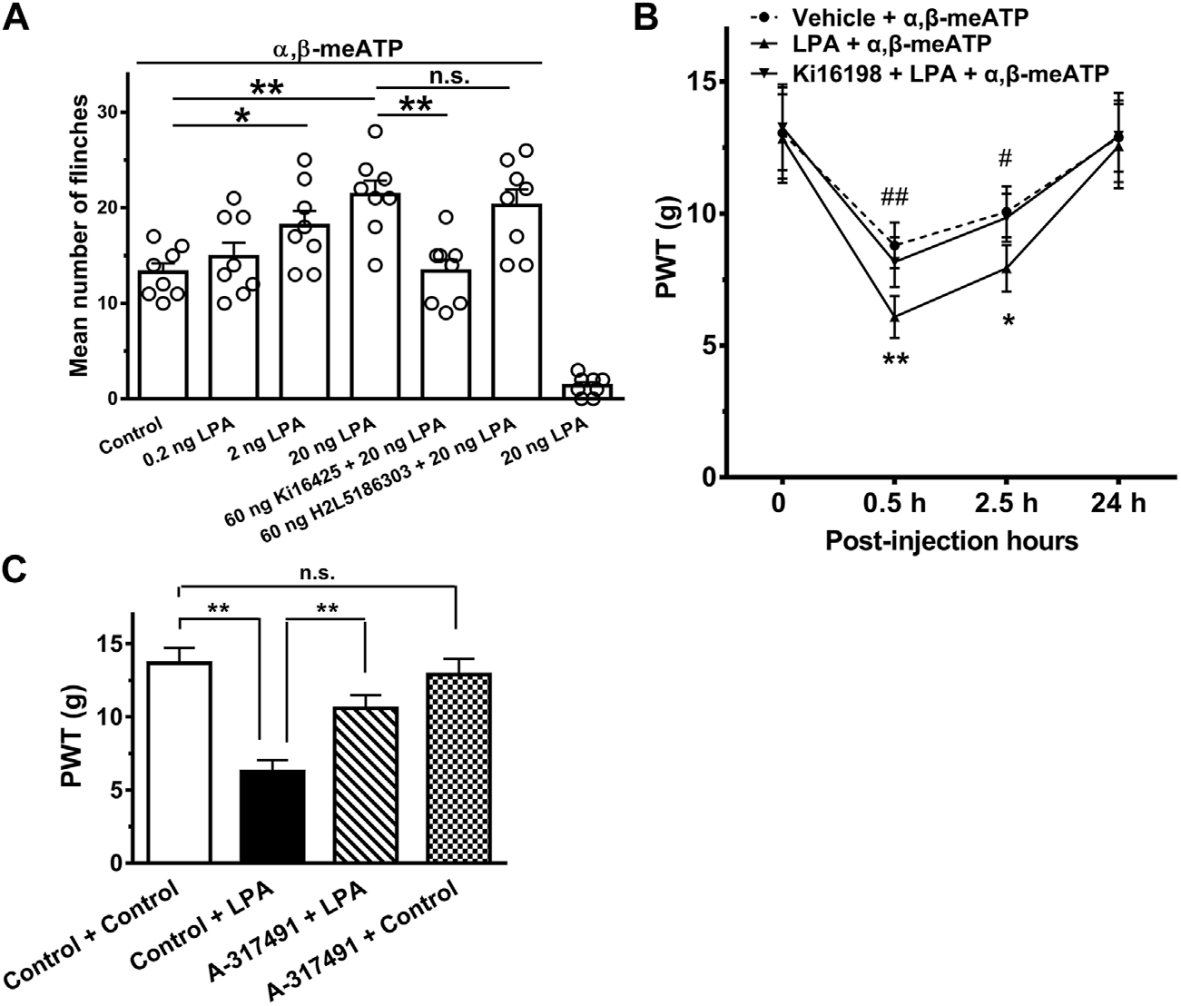
Exacerbation of α,β-meATP-induced nociceptive behaviors by LPA in rats. **(A)** Rats displayed spontaneous flinching responses after α,β-meATP (30 μg in 50 μl) was injected into the hind paws. LPA (0.2, 2 and 20 ng) increased the number of α,β-meATP-evoked flinching behaviors in dose-dependent manner when it was pre-treated into ipsilateral hind paws. Potentiation of the flinching behaviors by LPA (20 ng) was blocked by co-treated 60 ng the LPA_1_ receptor antagonist Ki16198, but not by co-treated 60 ng the LPA_2_ receptor antagonist H2L5185303. In addition, 20 ng LPA did not cause flinching behaviors. Bonferroni’s *post hoc* test, *p < 0.05, **p < 0.01; n.s., no significant. Each column represents the mean ± S.E.M. of 8 rats. **(B)** Intraplantar injection of α,β-meATP (30 μg in 50 μl) also caused a remarkable decrease in paw withdrawal thresholds (PWT, in g) at 0.5 and 2.5 h after injection and recovery at 24 h. The α,β-meATP-induced mechanical allodynia was significantly exacerbated by intraplantar pretreatment of LPA (20 ng), but not co-treatment of LPA (20 ng) and Ki16198 (60 ng). *p < 0.05, **p < 0.01, compared with Vehicle + α,β-meATP; #*p* < 0.05, ##*p* < 0.01, compared with LPA + α,β-meATP; Bonferroni’s *post hoc* test, *n* = 8 rats in each group. **(C)** Injections of LPA (i.t., 20 ug in 50 ul) decreased PWT, and this was attenuated by i.t. administration of A-317491 (i.t., 5 ug in 50 ul). In contrast, i.t. administration of A-317491 alone did not change the mechanical allodynia in rats. **p < 0.01; n.s., no significance. Each column represents the mean ± S.E.M. of 8 rats.

We finally observed whether potentiation of P2X3 receptors by LPA *in vitro* contributes to α,β-meATP-evoked nociceptive behaviors *in vivo*. Rats displayed spontaneous flinching/shaking responses when α,β-meATP (30 μg in 50 μl) was injected into the hind paws (Figure 5A). The α,β-meATP-evoked nociceptive behaviors was exacerbated in rats intraplantarly pretreated with LPA. Quantitative analysis showed that LPA dose-dependently (0.2, 2 and 20 ng) increased the number of flinching events induced by α,β-meATP (*p* < 0.05 and 0.01, one-way ANOVA followed by *post hoc* Bonferroni’s test, *n* = 8). Figure 5A shows that the exacerbation of nociceptive behaviors by 20 ng LPA was blocked by co-treated 60 ng the LPA_1_ receptor antagonist Ki16198 (*p* < 0.01, one-way ANOVA followed by *post hoc* Bonferroni’s test, *n* = 8), but not by co-treated 60 ng the LPA_2_ receptor antagonist H2L5185303. In addition, intraplantar injection of 20 ng LPA alone did not cause flinching behaviors.

We also observe that the effect of LPA on the mechanical allodynia induced by α,β-meATP in rats. Figure 5B shows intraplantar injection of α,β-meATP (30 μg in 50 μl) resulted in a significant decrease in the paw withdrawal threshold (PWT) within 0.5 and 4 h after injection, and recovery at 24 h. Intraplantar pretreatment of LPA had also an aggravating effect on the mechanical allodynia. The α,β-meATP-induced mechanical allodynia was significantly aggravated within 0.5 and 4 h after 20 ng LPA pretreatment (*p* < 0.05 and 0.01, Bonferroni’s *post hoc* test, compared with vehicle + α,β-meATP group, *n* = 8 rats; Figure 5B). The aggravating effect of LPA was completely blocked by co-treated 60 ng Ki16198 (*p* < 0.05 and 0.01, Bonferroni’s *post hoc* test, compared with LPA + α,β-meATP group, *n* = 10 rats; Figure 5B).

Administration of LPA (i.t.) has been shown to induced mechanical allodynia (Pan et al., 2016). To further address the specific role of P2X3 receptors in LPA-induced potentiation of pain behaviors, we observed that LPA-induced mechanical allodynia after pharmacological blockade of P2X3 receptors. As shown in Figure 5Ci.t. administration of A-317491 (5 ug in 50 ul), a specifical P2X3 and P2X2/3 receptor antagonist, significantly decreased the mechanical allodynia induced by LPA (i.t., 20 ug in 50 ul). In contrast, i.t. administration of A-317491 alone did not change the mechanical allodynia in rats.

Together, the results suggest that LPA also exacerbated α,β-meATP-evoked nociceptive behaviors in rats by activating peripheral LPA_1_ receptors rather than LPA_2_ receptors.

## Discussion

The present data demonstrated that LPA could enhance the functional activity of P2X3 receptors. LPA enhanced α,β-meATP-evoked electrophysiological activity in rat DRG neurons, which was involved LPA_1_ receptors, G-proteins and PKC signaling cascades. Behaviorally, LPA also exacerbated α,β-meATP-induced nociceptive behaviors in rats by activating peripheral LPA_1_ receptors.

In the present experiments, α,β-meATP-induced ATP currents were blocked by specific antagonist of P2X3 and P2X2/3 receptors, but not by antagonists of P2X4 receptors and P2X7 receptors, indicating they were P2X3 or P2X3-containing receptor-mediated currents. Moreover, α,β-meATP is only an activator of P2X3 and P2X1 receptors (North, 2002). P2X3 homomeric and P2X2/3 heteromeric receptors have been shown to be the most prevalent isoforms in sensory neurons, especially in a subset of small- and medium-sized nociceptive neurons (Vulchanova et al., 1997; Bradbury et al., 1998; Ueno et al., 1999; Dunn et al., 2001; Teixeira et al., 2017). The present study showed that LPA had potentiating effects on ATP currents, which were depended on concentration of LPA, but not clamping potentials. The LPA-induced potentiation involved an increase in the maximum response to α,β-meATP, but not the changes in affinity. Under the current-clamp conditions, LPA increased membrane excitability induced by α,β-meATP in DRG cells, including APs and membrane depolarization. Obviously, the results of current- and voltage-clamp confirmed each other.

Among the LPA_1-6_ receptor subtypes, LPA_1_ receptor is the major subtype located in primary sensory neurons and involved in pain (Inoue et al., 2004; Xie et al., 2008). Ki16198, a LPA_1_ receptor antagonist, blocked the enhancement of α,β-meATP-evoked ATP currents and nociceptive behaviors by LPA, suggesting that LPA sensitized P2X3 receptors through LPA_1_ receptors. In contrast, the LPA_2_ receptor antagonist H2L5185303 had little effect on LPA-induced potentiation, suggesting no involvement of LPA_2_ receptors. Immunofluorescent assays have shown that LPA_1_ receptor and P2X3 receptor are co-localized in DRG cells (Wu et al., 2016). Consistent with the present results, LPA increases Nav1.8 currents and TRPV1 currents in DRG neurons by activating LPA_1_ receptors in DRG cells (Pan et al., 2010; Pan et al., 2016).

**FIGURE 6:**
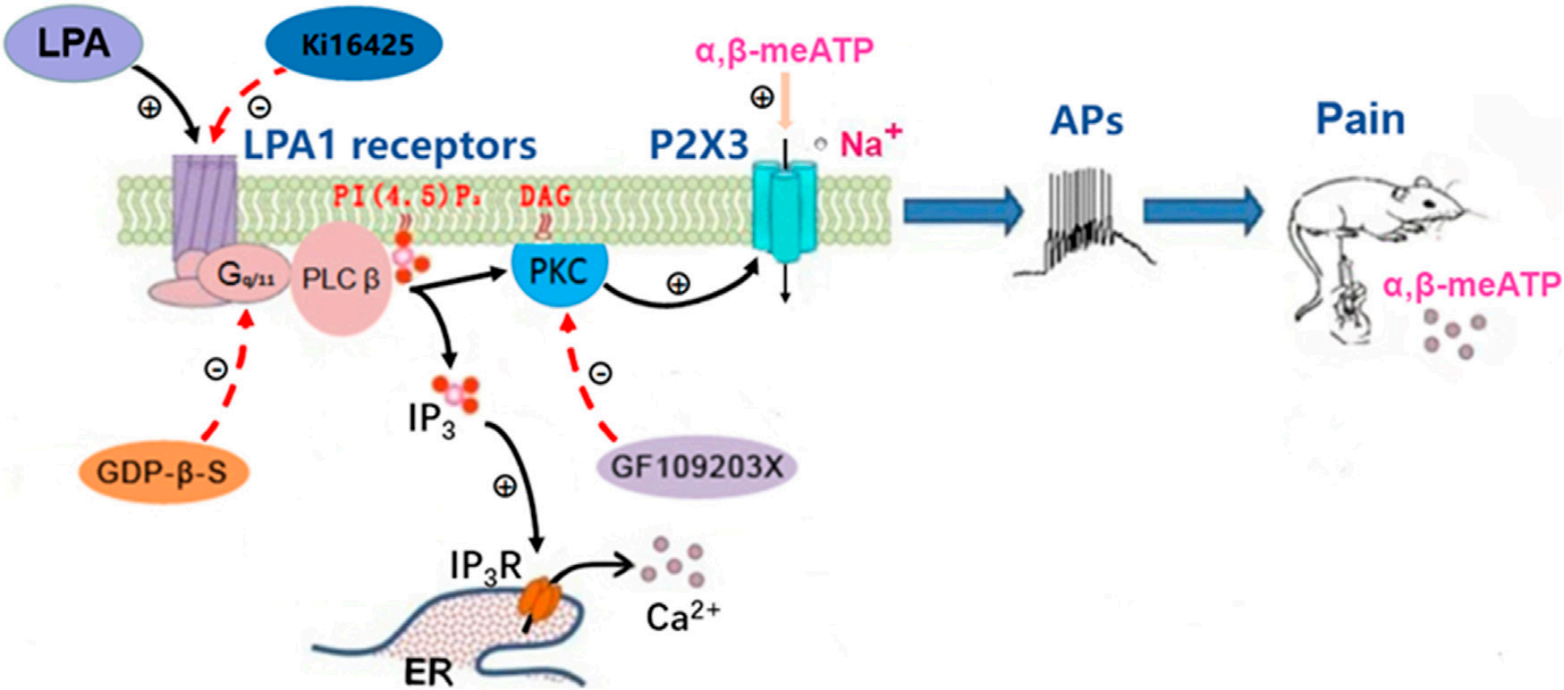
Schematic drawing of the modulation of P2X3 receptors by LPA. Left: The LPA_1_ receptor couples to heterotrimeric G_q/11_ proteins to activate PLCβ. PLCβ hydrolyzes membrane PI (4, 5) P2 to IP3 and DAG. DAG activates PKC, which enhances the activity of P2X3 receptors. LPA activates LPA_1_ receptors, resulting in the potentiation of P2X3 receptor activity *via* the above signal pathway, while the potentiation was blocked by inhibition of LPA_1_ receptors, Gq/11 proteins and PKC signaling by Ki16198, GDP-β-S and GF109203X. Middle: Activation of P2X3 receptors by α,β-meATP causes an inward-current, which drives nociceptors to the threshold and initiates action potentials (APs). Right: Intraplantar injection of α,β-meATP activates P2X3 receptors in peripheral nociceptors to produce nociceptive information, which is transmitted to the brain and then elicits pain. The LPA potentiation of α,β-meATP-activated currents in DRG neurons resulted in an increase of action potentials number and then an amplification of α,β-meATP -induced pain.

LPA_1_ receptor couples to four distinct G_α_ proteins (G_i/o_, G_q/11_, G_12/13_ and G_s_), to regulate intracellular signaling (Anliker and Chun, 2004; Yung et al., 2014). LPA_1_ receptor activates the Rho cascades by coupling to G_12/13_ proteins, which can result in up-regulation of expression of several ion channels, such as P2X3 receptors, Ca_v_3.2 and Ca_v_α2δ1 channels (Inoue et al., 2004; Wu et al., 2016). LPA_1_ receptor activation contributes to neuropathic pain *via* a Rho-Rho kinase signaling pathway (Inoue et al., 2004). However, our data indicated Rho signaling did not play a role in sensitization of P2X3 receptors by LPA, since blockade of the Rho signaling had no effect on the enhancement of ATP currents by LPA. The Rho-ROCK pathway is involved in the P2X3 expression in bone cancer model (Wu et al., 2016). However, the Rho-ROCK pathway did not affect the function of P2X3 receptors. One possible explanation was that the signaling molecules of this pathway cannot acutely regulate P2X3 receptors. LPA_1_ receptor can also activate PKC signaling pathway by coupling to G_q/11_ proteins, resulting in an increase in Nav1.8 expression and Nav1.8 currents (Seung Lee et al., 2005; Pan et al., 2016). PKC signaling pathway, rather than Rho pathway, has been shown to mediate potentiation of TRPV1 currents by activation of LPA_1_ receptors in DRG neurons (Pan et al., 2010). The present study showed that LPA-induced enhancement of ATP currents was completely prevented by intracellular application of GF-109203X, a selective PKC inhibitor, indicating involvement of PKC signaling. The α,β-meATP-induced currents are reported to be potentiated by PKC signaling in rat DRG neurons (Wang et al., 2012; Jin et al., 2020). Thus, PKC signaling rather than Rho signaling was involved in LPA-induced enhancement of ATP currents (Figure 6).

P2X3 receptor is also expressed in peripheral nociceptive sensory nerve endings, along with the soma of DRG neurons. P2X3 receptor belongs to cation channel. Once activated, it produces inward currents, which is sufficient to cause membrane potential depolarization and even bursts of APs (Xu et al., 2012). ATP activates P2X3 receptors and causes pain when injected into the skin (Cockayne et al., 2000). α,β-meATP also produces spontaneous nociceptive behaviors when injected into rat hindpaws, such as licking, lifting and biting of the injected paws, which significantly blocked by the P2X3 receptor antagonist A-317491 (McGaraughty et al., 2003). In rats with bone cancer, blockade of LPA_1_ receptors could inhibit α, β-meATP-induced spontaneous pain (Wu et al., 2016). The current behavioral results showed that peripheral preapplication of LPA exacerbated the α,β-meATP-triggered nociceptive behaviors in dose-dependent manner. The exacerbation of LPA occurred locally through direct activation of peripheral LPA_1_ receptors, but not through LPA_2_ receptors. These behavioral findings apparently confirmed the aforementioned electrophysiological results that LPA potentiated ATP currents in rat DRG neurons through LPA_1_ receptors. Considering PKC and intracellular Ca^2+^ concentration can also regulate other ion channels, we cannot rule out that LPA aggravated α,β-meATP-induced nociceptive behaviors through these ion channels. But current data LPA/A1 receptor signaling sensitized P2X3 receptors indicated that P2X3 receptors were involved in the modulation of α,β-meATP-induced nociceptive behaviors by LPA, at least partially. In addition, pharmacological blockade of P2X3 receptors by A-317491 significantly decreased the mechanical allodynia induced by i.t. administration of LPA, further indicating the specific role of P2X3 receptors in LPA-induced potentiation of pain behaviors.

The enhancement of P2X3 receptor activity by LPA may have pathophysiological significance. Under pathological conditions, such as tissue injury and inflammation, both LPA and ATP signaling may appear together. Peripherally, ATP can be released from inflammatory cells and injured tissues as an “injury” signaling, resulting in a nociceptive response by directly activating P2X3-containing receptors located in nociceptors (Cook et al., 1997; Cook and McCleskey, 2002; Kato et al., 2017). LPA is also an endogenous lipid metabolite and further released during tissue injury or inflammation states (Eichholtz et al., 1993). LPA levels are elevated to 0.1–1 µM in inflammatory exudates (Frisca et al., 2012). The LPA levels are increased in the synovial fluid of patients with knee osteoarthritis (McDougall et al., 2017). The released LPA activates LPA_1_ receptors, resulting in increased expression of P2X3 receptors *via* a Rho-ROCK pathway (Wu et al., 2016). Moreover, activation of LPA_1_ receptors by LPA enhances the functional activity of P2X3 receptors *via* a PKC signaling pathway. Current electrophysiological recordings took the soma of DRG cells as a model to reflect the characteristics of peripheral nerve endings. Activation of LPA_1_ receptors by LPA may increase P2X3 receptor-mediated currents and action potential bursts by sensitizing co-existed P2X3 receptors located on the nociceptive sensory terminals, then resulting in exacerbated nociceptive behaviors in rats (Figure 6).

## Conclusion

Our results further indicate that P2X3 receptor was a target of LPA, contributing to nociceptive behaviors. Beside an increase in the expression of P2X3 receptors *via* a Rho-ROCK pathway (Wu et al., 2016), LPA also enhanced the functional activity of P2X3 receptors in rat nociceptive DRG neurons *via* a LPA_1_ receptor and its downstream PKC signaling pathway. These findings provided a novel peripheral mechanism underlying the sensitization of pain.

## Data Availability

The raw data supporting the conclusion of this article will be made available by the authors, without undue reservation.

## Abbreviations

α,β-meATP, α,β-methylene-ATP; ANOVA, analysis of variance; DMEM, Dulbecco’s modified Eagle’s medium; DRG, dorsal root ganglia; EC50, half-maximal effective concentration; GPCRs, G protein-coupled receptors; I_ATP_, ATP current; LPA, Lysophosphatidic acid; PKC, protein kinase C; TRPV1, transient receptor potential V1.
